# Host deficiency in ephrin-A1 inhibits breast cancer metastasis

**DOI:** 10.12688/f1000research.22689.2

**Published:** 2020-05-14

**Authors:** Eileen Shiuan, Ashwin Inala, Shan Wang, Wenqiang Song, Victoria Youngblood, Jin Chen, Dana M. Brantley-Sieders

**Affiliations:** 1 Program in Cancer Biology, Vanderbilt University, Nashville, TN, 37232, USA; 2 Medical Scientist Training Program, Vanderbilt University, Nashville, TN, 37232, USA; 3 Veterans Affairs Medical Center, Tennessee Valley Healthcare System, Nashville, TN, 37212, USA; 4 Division of Rheumatology and Immunology, Vanderbilt University Medical Center, Nashville, TN, 37232, USA; 5 School of Nursing, Duke University, Durham, NC, 27710, USA; 6 Vanderbilt-Ingram Cancer Center, Vanderbilt University Medical Center, Nashville, TN, 37232, USA; 7 Department of Cell and Developmental Biology, Vanderbilt University, Nashville, TN, 37232, USA

**Keywords:** Ephrin-A1, host-tumor interactions, breast cancer, metastasis, metastatic niche

## Abstract

**Background:** The conventional dogma of treating cancer by focusing on the elimination of tumor cells has been recently refined to include consideration of the tumor microenvironment, which includes host stromal cells. Ephrin-A1, a cell surface protein involved in adhesion and migration, has been shown to be tumor suppressive in the context of the cancer cell. However, its role in the host has not been fully investigated. Here, we examine how ephrin-A1 host deficiency affects cancer growth and metastasis in a murine model of breast cancer.

**Methods:** 4T1 cells were orthotopically implanted into the mammary fat pads or injected into the tail veins of ephrin-A1 wild-type ( *Efna1* ^+/+^), heterozygous ( *Efna1* ^+/-^), or knockout ( *Efna1* ^-/-^) mice. Tumor growth, lung metastasis, and tumor recurrence after surgical resection were measured. Flow cytometry and immunohistochemistry (IHC) were used to analyze various cell populations in primary tumors and tumor-bearing lungs.

**Results:** While primary tumor growth did not differ between *Efna1* ^+/+^, *Efna1* ^+/-^, and *Efna1* ^-/-^ mice, lung metastasis and primary tumor recurrence were significantly decreased in knockout mice. *Efna1* ^-/-^ mice had reduced lung colonization of 4T1 cells compared to *Efna1* ^+/+^ littermate controls as early as 24 hours after tail vein injection. Furthermore, established lung lesions in *Efna1* ^-/-^ mice had reduced proliferation compared to those in *Efna1* ^+/+^ controls.

**Conclusions:** Our studies demonstrate that host deficiency of ephrin-A1 does not impact primary tumor growth but does affect metastasis by providing a less favorable metastatic niche for cancer cell colonization and growth. Elucidating the mechanisms by which host ephrin-A1 impacts cancer relapse and metastasis may shed new light on novel therapeutic strategies.

## Introduction

Over the past several decades, the conventional dogma of treating cancer by focusing on the elimination of rapidly dividing tumor cells has been gradually refined to include consideration of the environment in which the tumor thrives – the tumor microenvironment. The tumor microenvironment consists of both cancer cells and host stromal cells, such as endothelial cells, immune populations, and fibroblasts. Prominent discoveries regarding tumor-associated endothelium and immune cells have notably led to breakthrough therapeutic strategies with anti-angiogenic agents and immunotherapies, respectively ^1– 4^. Thus, understanding the host-tumor interactions involved in tumor growth and metastasis is critical for the development and application of new anti-cancer therapies.

As a result of new advancements in targeted and immunotherapies, the majority of patients with early stage disease have a very favorable prognosis. However, patients who later develop distant metastasis or who are diagnosed with disseminated disease at the onset are typically very difficult to treat effectively ^5, 6^. This is largely because our knowledge of how cancer cells spread is still limited. Cancer metastasis is a dynamic and complex process that requires tumor cells to undergo many steps, including adopting invasive properties, intravasating into proximal vasculature, surviving in circulation, evading immunosurveillance, extravasating from distant vasculature, and finally adapting to selective pressures of a new environment ^7, 8^. Each of these steps involves multiple interactions between cancer cells and different types of host stromal cells. As an example, breast cancer most commonly metastasizes to the lung, bone, liver, and brain, but how and why these cells travel and colonize these particular organs is still unknown ^5, 6^. A better understanding of how breast cancer metastasizes to these distant sites is greatly needed in order to develop more effective therapies and prevent spread of malignant disease.

Ephrin-A1 is a cell surface protein that regulates cell adhesion and migration ^9– 28^, and its role in cancer has recently been investigated in several different solid tumors ^29– 35^. It belongs to the group of ephrin ligands that interact with the largest family of receptor tyrosine kinases (RTKs), the Eph receptors, and regulates various developmental processes, such as embryonic cardiovascular development and angiogenic remodeling ^36– 38^. It is expressed in various cell types, including epithelial, endothelial, and immune cells and is the primary ligand for EphA2 RTK, which has been implicated in cancer growth and metastasis in various solid tumors ^36– 41^. While ephrin-A1 expression in cancer cells has been shown to be tumor suppressive ^25, 26, 42,^, its role in the host, has not been fully investigated. Here, we use ephrin-A1 knockout mice to examine how ephrin-A1 host deficiency affects cancer growth and metastasis in a murine model of breast cancer.

To test the impact of ephrin-A1 host deficiency on cancer progression, we utilized an orthotopic 4T1 mammary tumor model, as well as two different models of metastasis. While primary tumor growth did not significantly differ between ephrin-A1 wild-type ( *Efna1* ^+/+^), heterozygous ( *Efna1* ^+/-^), and knockout ( *Efna1* ^-/-^) mice, metastasis and primary tumor recurrence were significantly decreased in *Efna1* ^-/-^ mice. Results of analysis on tumor-infiltrating immune cell populations and vascularity in the primary tumor did not evidently explain the differences in metastasis between *Efna1* ^+/+^ and *Efna1* ^-/-^ mice. However, tumor cell lung colonization was reduced in *Efna1* ^-/-^ mice, and lung metastases in *Efna1* ^-/-^ mice were less proliferative than in their wild-type counterparts, suggesting that the metastatic niche in *Efna1* ^-/-^ mice is less hospitable for invading tumor cells. Together, our studies suggest that host deficiency of ephrin-A1 does not impact initial tumor growth but does affect metastasis through inhibiting cancer cell extravasation and proliferation at the metastatic niche.

## Methods

### Animal models

Animals were housed in a non-barrier animal facility under pathogen-free conditions, 12-hour light/dark cycle, and access to standard rodent diet and water *ad libitum*. Experiments were performed in accordance with AAALAC guidelines and with Vanderbilt University Medical Center Institutional Animal Care and Use Committee approval. All mice used in this study were immunocompetent BALB/c mice. Ephrin-A1 knockout ( *Efna1* ^-/-^) mice were previously characterized by our lab ^43^. To generate littermate controls, wild-type BALB/c mice were purchased from Jackson Laboratory and mated with *Efna1* ^-/-^ mice to generate heterozygote mating pairs. *Efna1* ^+/+^, *Efna1* ^+/-^ and *Efna1* ^-/-^ animals were identified by PCR analysis of genomic DNA using the following primers: Forward primer (5’-TGGTTATATCCCCCCACCTCACAC-3’) and two allele-specific reverse primers (WT 5’-AAGGACTCCCATATCTCAGCGACG-3’) and (KO 5’-AGACTGCCTTGGGAAAAGCG-3’). Mice were co-housed with one to four littermates for at least two weeks prior to and during all experiments and compared with littermate controls whenever possible. All mice used for tumor experiments were six to ten weeks old at the onset on the experiment. Experimental cohorts were limited to litters that were born within two consecutive weeks and that also had at least one *Efna1* ^-/-^ and *Efna1* ^+/+^ female littermate pair and, when applicable, at least one *Efna1* ^+/-^ female littermate. Sample sizes are as shown in the figures and range from three to twelve mice per group. At experimental endpoints, mice were euthanized by cervical dislocation.

### Cell culture

4T1 murine mammary adenocarcinoma cells were purchased from ATCC and maintained in DMEM (Corning #MT10013CV) supplemented with penicillin/streptomycin (Gibco #15140163) and 10% FBS (Gibco #A3160502). 4T1-GFP-luciferase clones were generated by serial dilutions of 4T1 cells with lentiviral overexpression of GFP and luciferase genes.

### Tumor models

To reflect human breast cancer, only female mice were used for tumor experiments. For orthotopic mammary tumor implantations, 1×10 ^5^ 4T1 cells suspended in a 1:1 mixture of PBS and Growth Factor-Reduced Matrigel (Corning #354230) in a total volume of 100 μl were injected through the nipple into the fourth mammary fat pads of six to eight-week-old female mice. Tumor dimensions were measured by digital caliper at given time points every other day, and volume was calculated using the following formula: volume = length × width ^2^ × 0.52. To observe spontaneous lung metastases and primary tumor recurrence, mammary tumors were resected at day 14 post-implantation, along with draining inguinal lymph nodes and surrounding fat pads, and mice were ultimately sacrificed at day 32. At the time of surgical resection of primary tumors on day 14, tumors were weighed and cut in half to provide tissue for both flow cytometry analysis and cryosection staining. At the experimental endpoint on day 32, tumors were weighed, and lung metastases were counted in a blinded manner. For lung colonization experiments, 4T1-GFP-luciferase cells suspended in PBS were injected via tail vein, and mice underwent *in vivo* bioluminescence imaging with a PerkinElmer IVIS Spectrum several hours post-injection to verify successful and equal delivery of 4T1 cells. To observe gradual formation of GFP+ metastases, 1×10 ^5^ 4T1-GFP-luciferase cells were injected via tail vein, and mice were sacrificed at day 17. GFP+ lung metastases were counted in a blinded manner. The left lung lobe of each mouse was fixed in 10% formalin for subsequent formalin-fixed paraffin-embedded (FFPE) processing, sectioning, and H&E staining, while the other lung lobes were processed for flow cytometry analysis. To observe early colonization and proliferation of 4T1 cells, 5×10 ^5^ 4T1-GFP-luciferase cells labeled with CellTrace Violet dye (Invitrogen #C34571) were injected via tail vein. At 24 hours, mice were sacrificed, and lungs were perfused with PBS and processed for flow cytometry analysis.

### Immunohistochemistry (IHC) and Immunofluorescence (IF)

FFPE lung sections were prepared and stained for PCNA (1:100, BD Biosciences #555567 raised in mouse, RRID: AB_395947) as described previously ^44^. Slides were blinded, and the number of metastatic foci per section of lobe was quantified. Nuclear PCNA staining was analyzed using ImageJ v1.52o with the IHC Profiler plugin ^45^ and percentage of PCNA+ tumor cell nuclei were quantified. Each data point is an average of two sections of the left lung from an individual mouse. To prepare cryosections, mammary tumors were frozen in OCT Compound (Thermo Fisher Scientific #23-730-571) on dry ice and stored at -80°C. Sections (8 µm) were cut on a Leica Cryostat CM1950, fixed in 4% PFA, washed with PBS, permeabilized with 0.3% Triton X-100 (Sigma-Aldrich #X100), and blocked using M.O.M. Mouse Ig Blocking Reagent and Protein Concentrate (Vector Laboratories #PK-2200) per manufacturer recommendations and with 2.5% goat serum (Sigma-Aldrich #G9023) in PBS. Slides were then incubated over two nights at 4°C with primary antibodies against CD31 (1:150, Biolegend #102501 raised in rat, RRID: AB_312908) and αSMA (1:150, Dako #M085129-2 raised in mouse, RRID: AB_2811108) in blocking buffer. After washing with PBS, slides were incubated for one hour at room temperature in secondary antibodies goat anti-rat Ax594 (1:500, Invitrogen #A11007, RRID: AB_10561522) and anti-mouse Ax488 (1:500, Invitrogen #A11001, RRID: AB_2534069), washed with PBS, and mounted with ProLong Gold Antifade Mountant with DAPI (Invitrogen #P36931). Slides were blinded, and images were taken by an Olympus DP72 camera through a BX60 inverted fluorescence microscope and processed using CellSens Dimension software. A total of 12-40 20x fields of view were analyzed from each section using ImageJ. For αSMA analysis, images were evaluated for colocalization with CD31 staining, and data was displayed as a percentage of αSMA+ out of CD31+ area or integrated intensity. Each data point is an average of all fields of view of two to three tumor sections from an individual mouse.

### Flow cytometry

Tumors and lungs were minced and dissociated in RPMI-1640 media (Corning #MT10040CV) containing 2.5% FBS, 1 mg/ml collagenase IA (Sigma-Aldrich #C9891), and 0.25 mg/ml DNase I (Sigma-Aldrich #DN25) for 45 minutes at 37°C. Digested tissue was then filtered through a 70-µm strainer, and red blood cells were lysed using ACK Lysis Buffer (KD Medical #RGF-3015). Samples were washed with PBS and stained with Ghost Dye Violet V510 (Tonbo Biosciences #13-0870) to exclude dead cells. After washing with buffer (0.5% BSA, 2mM EDTA in PBS), samples were blocked in αCD16/32 mouse Fc block (Tonbo Biosciences #70-0161) and stained for extracellular proteins using an antibody master mix made in buffer. After washing with buffer, cells were fixed with 2% PFA. For FoxP3 intracellular staining, cells were permeabilized using the FoxP3 Transcription Factor Staining Kit (Tonbo Biosciences #TNB-0607-KIT) per manufacturer protocol. Flow cytometry data was obtained on a BD 4-laser Fortessa using BD FACS Diva software v8.0.1 and analyzed using FlowJo software v10.6.1. Fluorescence minus one (FMO) samples were used as gating controls when needed. Antibodies used in flow panels are detailed in Table 1, and gating strategies used in analysis are detailed in Table 2. Each data point is generated after analyzing at least 5×10 ^5^ viable cells from a specimen from an individual mouse.

**Table 1.** Antibodies used in flow cytometry analysis.

Antibody target	Manufacturer	Catalog #	Fluorophore	Dilution	RRID
MHCII I-E/A	Tonbo Biosciences	75-5321	V450	1/250	AB_2621965
CD8a	BD Biosciences	560469	V450	1/250	AB_1645281
CD11b	Tonbo Biosciences	35-0112	FITC	1/250	AB_2621676
CD62L	Tonbo Biosciences	35-0621	FITC	1/100	AB_2621697
CD44	Tonbo Biosciences	50-0441	PE	1/5000	AB_2621762
CTLA-4	BD Biosciences	561718	PE	1/250	AB_10895585
CD31	BD Biosciences	561073	PE	1/750	AB_10563931
CD4	Biolegend	100516	APC	1/1000	AB_312719
Ly6C	BD Biosciences	560595	APC	1/500	AB_1727554
FoxP3	eBiosciences	50-5773-82	e660	1/100	AB_11218868
F4/80	eBiosciences	45-4801-82	PerCP-Cy5.5	1/250	AB_914345
CD3e	Tonbo Biosciences	65-0031	PerCP-Cy5.5	1/250	AB_2621872
Ly6G (Gr1)	Tonbo Biosciences	80-5931	rF710	1/1000	AB_2621999
CD8a	Tonbo Biosciences	80-0081	rF710	1/500	AB_2621977
PD-1	BD Biosciences	565815	APC-R700	1/500	AB_2739366
CD45	Biolegend	103109	PE-Cy5	1/5000	AB_312974
CD4	Tonbo Biosciences	55-0041	PE-Cy5	1/2500	AB_2621816
CD69	BD Biosciences	552879	PE-Cy5	1/1000	AB_394508
CD11b	Tonbo Biosciences	55-0112	PE-Cy5	1/5000	AB_2621818
EpCAM	Biolegend	118215	PE-Cy7	1/750	AB_1236477
CD11c	BD Biosciences	561022	PE-Cy7	1/500	AB_2033997
Ly6C	eBiosciences	25-5932-80	PE-Cy7	1/1000	AB_2573502
CD25	Tonbo Biosciences	60-0251	PE-Cy7	1/500	AB_2621843
CD11c	Biolegend	117323	APC-Cy7	1/500	AB_830646
CD45	BD Biosciences	557659	APC-Cy7	1/500	AB_396774

**Table 2.** Gating strategy used in flow cytometry analysis.

Cell population	Gating strategy
CD8 T cells	CD45+,CD3e+,CD4-,CD8a+
CD4 T cells	CD45+,CD3e+,CD4+,CD8a-
Tregs	CD45+,CD3e+,CD4+,CD8a-,CD25+,FoxP3+
Monocytes	CD45+,CD11b+,Ly6G-,Ly6C+,F4/80-
Macrophages	CD45+,CD11b+,Ly6G-,Ly6C-,F4/80+
Granulocytes	CD45+,CD11b+,Ly6G+,Ly6C-/+,F4/80-
Dendritic cells	CD45+,CD11c+,MHCII+,F4/80-
Endothelial cells	CD45-,GFP-,EpCAM-,CD31+
4T1 cells	CD45-,GFP+,CD31-

### Statistical analysis

All graphs and statistical analysis were completed using GraphPad Prism software v6.07. For comparisons between two groups, an unpaired Mann-Whitney *U*-test was performed. For comparisons between three groups, a Kruskal-Wallis *H*-test was performed, followed by post-hoc Mann-Whitney *U*-tests evaluating differences between the knockout and either the wild-type or heterozygote animals. A *P*-value less than 0.05 was considered statistically significant.

## Results

### Ephrin-A1-deficient hosts have reduced metastasis *in vivo*

**Figure 1.**
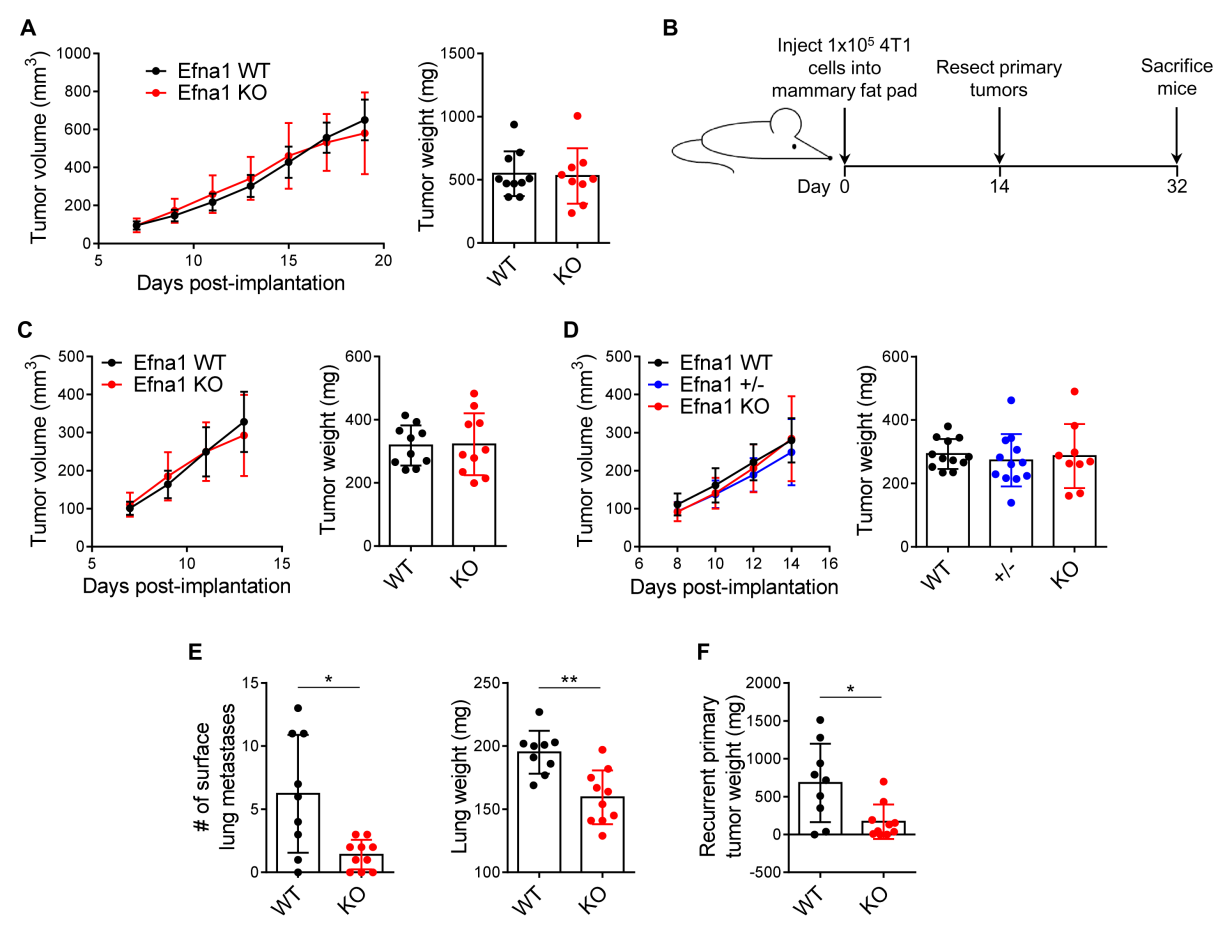
Ephrin-A1-deficient hosts have reduced metastasis and tumor recurrence but no difference in primary tumor growth. ( **A**) 4T1 primary tumor growth curves in age-matched female *Efna1* ^+/+^ (WT) and *Efna1* ^-/-^ (KO) mice and resulting tumor weights at day 21 post-implantation. ( **B**) Schematic diagram showing experimental procedure for evaluating spontaneous metastases. ( **C**) 4T1 primary tumor growth curves in WT and KO mice and resultant tumor weights at time of surgical resection on day 14. ( **D**) 4T1 primary tumor growth curves in WT, heterozygous (+/-) and KO littermates and resultant tumor weights at time of surgical resection on day 14. ( **E**) Blinded quantification of visible lung metastases and lung weights from WT and KO mice at experimental endpoint on day 32. ( **F**) Weights of recurrent 4T1 tumor at primary site 18 days after surgical resection. Data shown are averages ± SD with each data point representing an individual mouse ( *n*=9–12 mice per group). * *p*<0.05, ** *p*<0.01 (unpaired Mann-Whitney *U*-test).

We initially investigated the impact of ephrin-A1 host deficiency on primary tumor growth by implanting 4T1 cells in a mixture of PBS and Matrigel orthotopically into the mammary fat pads of syngeneic BALB/c female *Efna1* ^+/+^ and *Efna1* ^-/-^ mice. No difference in primary tumor growth or weight at 21 days post-implantation was observed ( Figure 1A). To test the impact of ephrin-A1 host deficiency on spontaneous metastasis, 4T1 cells were implanted orthotopically as described above and surgically resected on day 14 post-implantation to allow for gradual development of endogenous metastases by day 32 ( Figure 1B). As expected, primary tumors resected from *Efna1* ^+/+^ and *Efna1* ^-/-^ mice were not different in size ( Figure 1C), and this was additionally verified with *Efna1* ^+/+^, *Efna1* ^+/-^, and *Efna1* ^-/-^ littermates ( Figure 1D). However, at the experimental endpoint, the number of visible lung metastases and lung weights were significantly decreased in knockout mice ( Figure 1E). Many of these mice not only harbored lung metastases but also tumors that had regrown at the original site of the resected primary tumor. Similar to our findings in lung metastases, the size of recurrent primary tumors was significantly reduced in knockout mice ( Figure 1F). Together, these results demonstrate that while host deficiency in ephrin-A1 may not affect initial tumor growth, it can impact metastatic spread and recurrence. *Underlying data* are available ^46, 47^.

**Figure 2.**
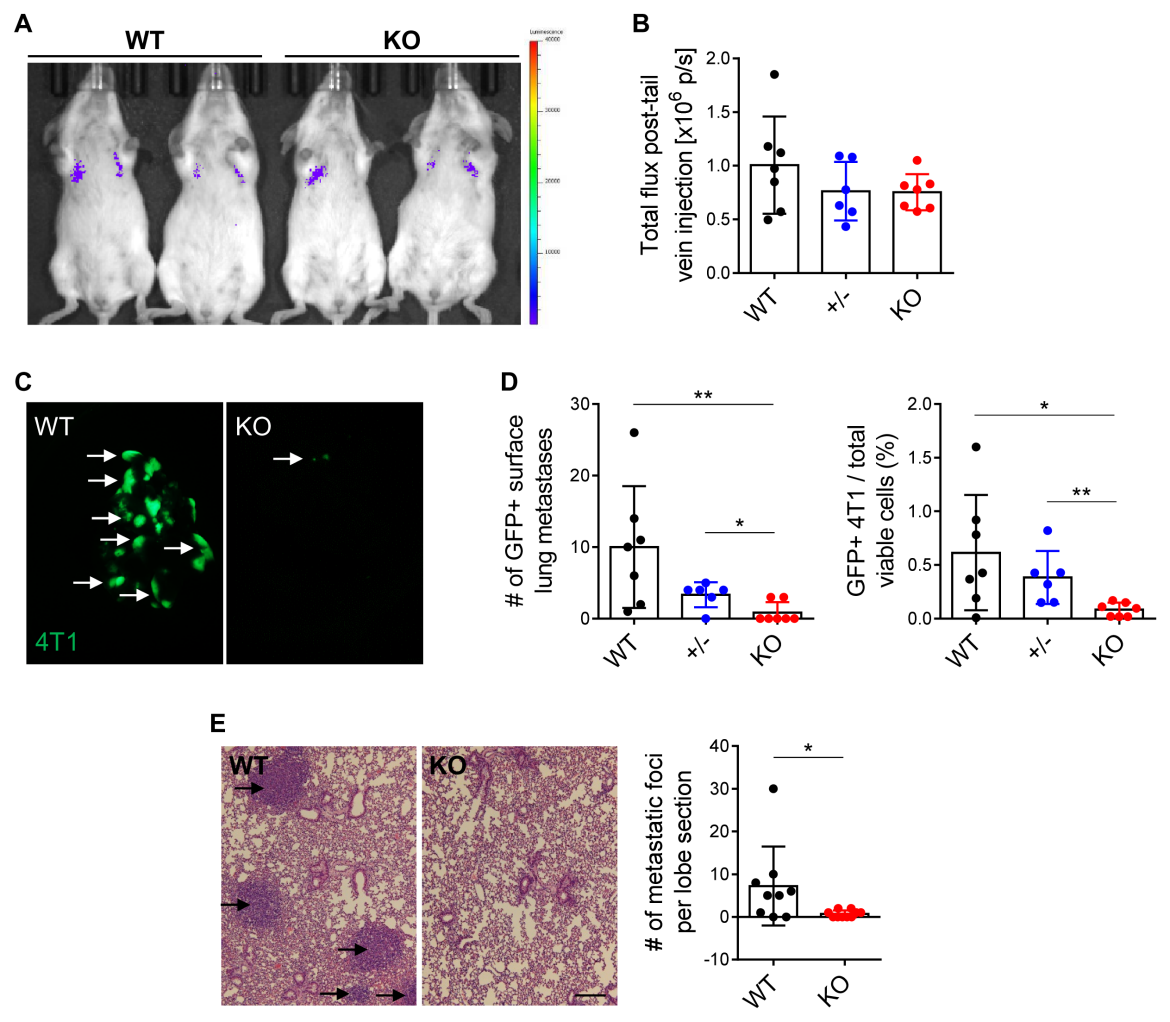
Ephrin-A1-deficient hosts have reduced cancer cell lung colonization. ( **A**) Representative image of bioluminescence signal in WT and KO littermates several hours after tail vein injection of 1×10 ^5^ 4T1-GFP-luciferase cells. ( **B**) Quantification of bioluminescence signal in WT, +/-, and KO littermates. ( **C**) Representative images of GFP+ surface lung metastases in WT and KO littermates 17 days after tail vein injection. ( **D**) Blinded quantification of GFP+ lung metastases in WT, +/-, and KO littermates and percentages of GFP+ 4T1 cells in the lung from flow cytometry analysis. ( **E**) Representative H&E staining of left lung lobes from WT and KO littermates and blinded quantification of metastatic foci per lung section. Scale bar: 200 µm. Data shown are averages ± SD with each data point representing an individual mouse ( *n*=4–9 mice per group). * *p*<0.05, ** *p*<0.01 (unpaired Mann-Whitney *U*-test for comparisons between two groups, Kruskal-Wallis *H*-test with post-hoc unpaired Mann-Whitney *U*-test for comparisons between three groups).

To complement our findings in our model of spontaneous metastasis, we evaluated the impact of ephrin-A1 host deficiency on experimental metastasis. 4T1 cells engineered to express GFP and luciferase (4T1-GFP-luciferase) were injected into the tail veins of *Efna1* ^+/+^, *Efna1* ^+/-^, and *Efna1* ^-/-^ littermates. *In vivo* bioluminescence imaging several hours after injection illustrated comparable signal across all mice ( Figure 2A, B), indicating ephrin-A1 host deficiency did not impact tumor cell trafficking and lodging within the lung, at least in this short time frame. After harvesting the lungs 17 days later, we observed decreased GFP+ metastases in *Efna1* ^-/-^ mice, compared to both *Efna1* ^+/+^ and *Efna1* ^+/-^ littermates, which was additionally confirmed by flow cytometry ( Figure 2C, D). Similarly, histological analysis revealed fewer metastatic foci in lungs from *Efna1* ^-/-^ mice ( Figure 2E). These data align with our previous observations on endogenous metastasis and suggest that host deficiency in ephrin-A1 inhibits circulating cancer cells from colonizing the lung. *Underlying data* are available ^48, 49^.

### Tumor-infiltrating immune populations are not significantly different in ephrin-A1-deficient hosts

Ephrin-A1 is expressed in several types of host cells, including immune cells and endothelial cells ^39– 41^. Thus, we sought to determine how the ephrin-A1-deficient host immune system and endothelium may mitigate metastasis. Among immune cells, Ephrin-A1 can be expressed in B and T cells, monocytes, and macrophages ^39^. The role of ephrin-A1 in B cells is largely unknown ^50^. However, in T cells, monocytes, and macrophages, ephrin-A1 has been shown to regulate cell adhesion and migration ^11, 12, 15, 27, 51– 54^. These immune cell populations play a critical role in overall anti-tumor immunity and immunosurveillance. Dendritic cells and T cells, particularly CD8 cytotoxic T cells, are the primary drivers of the adaptive anti-tumor response in solid tumors and increased infiltration of these cell types is correlated with better prognosis and enhanced response to immunotherapies ^55, 56^. Conversely, T regulatory cells (Tregs) suppress effector functions of T cells and typically inhibit the anti-tumor response ^57, 58^. Between the two ends of this spectrum, myeloid populations, such as monocytes, macrophages, and granulocytes, can either promote or suppress an anti-tumor response, depending on their polarization and functionality ^59, 60^.

**Figure 3.**
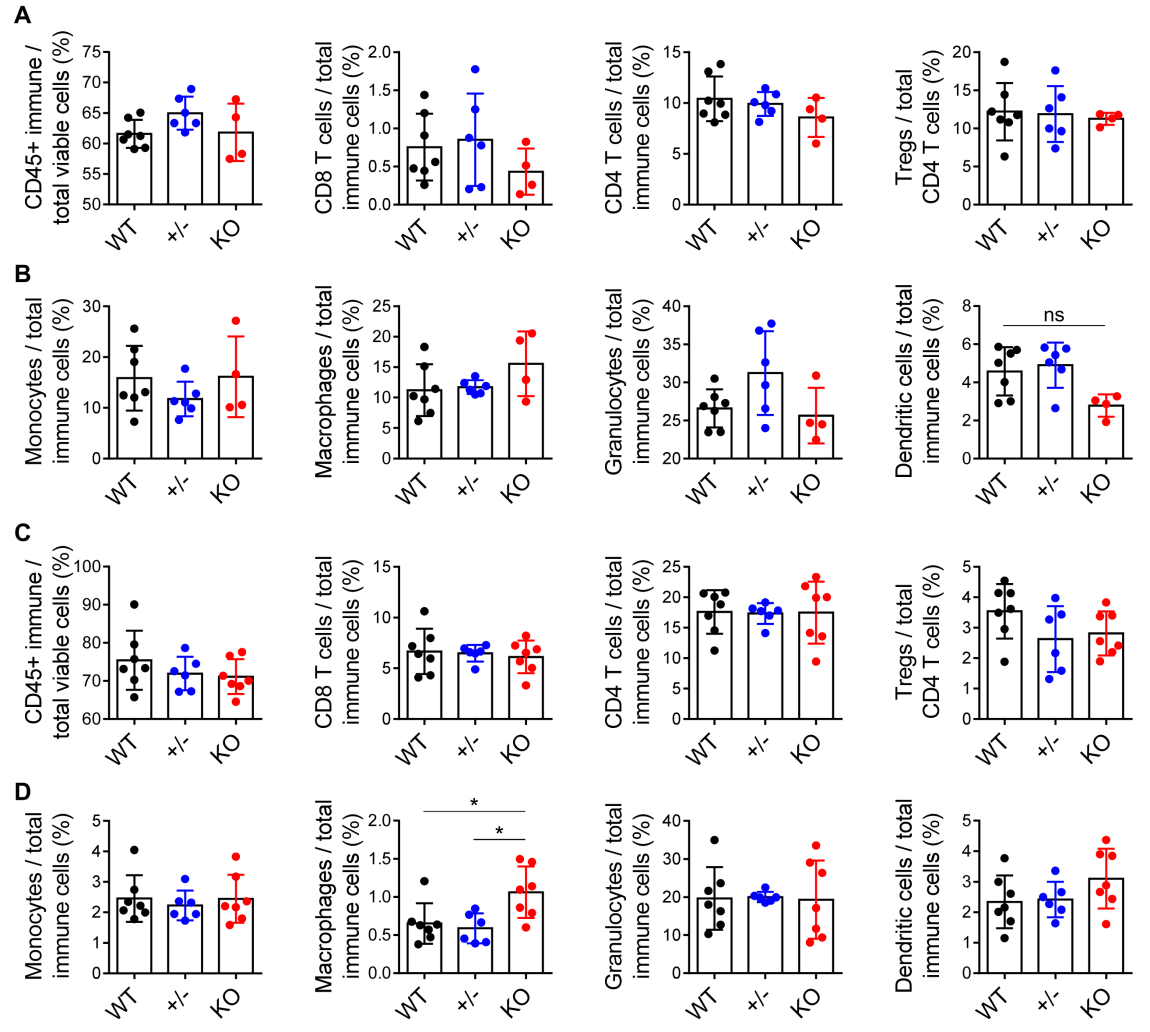
Tumor-infiltrating immune populations are not significantly different in ephrin-A1-deficient hosts. ( **A**) Flow cytometric analysis of total immune cells, T cells, and T regulatory (Treg) cells, as well as ( **B**) monocytes, macrophages, granulocytes, and dendritic cells, in 4T1 primary tumors resected from WT, +/-, and KO littermates at day 14 post-implantation. ( **C**, **D**) Similar analyses of immune populations in tumor-bearing lungs harvested from WT, +/-, and KO littermates 17 days after tail vein injection of 4T1-GFP-luciferase cells. Data shown are averages ± SD with each data point representing an individual mouse ( *n*=3–7 mice per group). * *p*<0.05 (Kruskal-Wallis *H*-test with post-hoc unpaired Mann-Whitney *U*-test).

Because of ephrin-A1’s known role in adhesion and chemotaxis of immune cells, we performed flow cytometry analysis on 4T1 primary tumors harvested from *Efna1* ^+/+^, *Efna1* ^+/-^, and *Efna* ^-/-^ littermates. To our surprise, we found no significant differences in total infiltrating immune cells, CD4 or CD8 T cells, dendritic cells, Tregs, or myeloid populations in *Efna1* ^+/+^, *Efna1* ^+/-^, and *Efna* ^-/-^ littermates ( Figure 3A, B). Dendritic cells were decreased in *Efna* ^-/-^ mice, though not significantly with these sample sizes. While there were no apparent differences in the immune microenvironment of the mammary tumors, the immune microenvironment of the lung is distinct from that of the mammary gland and may impact the metastatic niche. To investigate this, we performed flow cytometry analysis on 4T1 tumor-bearing lungs generated from our model of experimental metastasis. Similar to the results we obtained from the 4T1 primary tumors, we did not see significant differences in immune populations in tumor-bearing lungs harvested from *Efna1* ^+/+^, *Efna1* ^+/-^, and *Efna1* ^-/-^ littermates, except for a modest increase in macrophages in knockout mice ( Figure 3C, D). *Underlying data* are available ^61, 62^.

 Although the percentage of tumor infiltrating T cells in *Efna1* ^+/+^ and *Efna1* ^-/-^ mice is comparable, their activation status and effector function may still be different. Tumor-infiltrating T cells with upregulated expression of activation markers, such as CD44, CD69, and CD25, and downregulated expression of antigen-naïve markers like CD62L and exhaustion markers like PD-1 and CTLA-4 indicate a higher T cell functional status that mediates a stronger and more enduring anti-tumor response ^57, 58^. We assessed these markers on T cells in 4T1 primary tumors and tumor-bearing lungs from *Efna1* ^+/+^ and *Efna1* ^-/-^ littermates using flow cytometry. However, we did not observe consistent increases in activation or decreases in naïve or exhaustion markers in knockout-derived T cells (data not shown, included in *Underlying data*) ^61^. In summary, host deficiency in ephrin-A1 does not significantly affect tumor-infiltrating immune cells in both primary tumors and tumor-bearing lungs. Thus, the reduction of lung metastases in *Efna1* ^-/-^ hosts *in vivo* is unlikely due to host immunity.

### Tumor vascularity and pericyte coverage are not significantly different in ephrin-A1-deficient hosts

In addition to the anti-tumor immune response, another host factor that can impact metastasis is the tumor vasculature. Angiogenesis is the formation of new blood vessels from a pre-existing network and is required for solid tumor growth and progression. Blood vessels can supply nutrients that support tumor growth and provide an entry for hematological dissemination and invasion ^1, 63^. These new blood vessels are typically hastily constructed in response to the high release of growth factors, such as vascular endothelial growth factor (VEGF), from tumor cells ^64, 65^. Thus, tumor vessels tend to be disorganized, leaky, and poorly covered by pericytes, which normally support the integrity of the endothelium. Ephrin-A1 is expressed in the vascular endothelium and has been shown to promote angiogenesis *in vitro* and in several *in vivo* models ^66– 71^. Therefore, we hypothesized that tumors in *Efna1* ^-/-^ mice may have reduced tumor vasculature and increased endothelial pericyte coverage compared to *Efna1* ^+/+^ controls.

**Figure 4.**
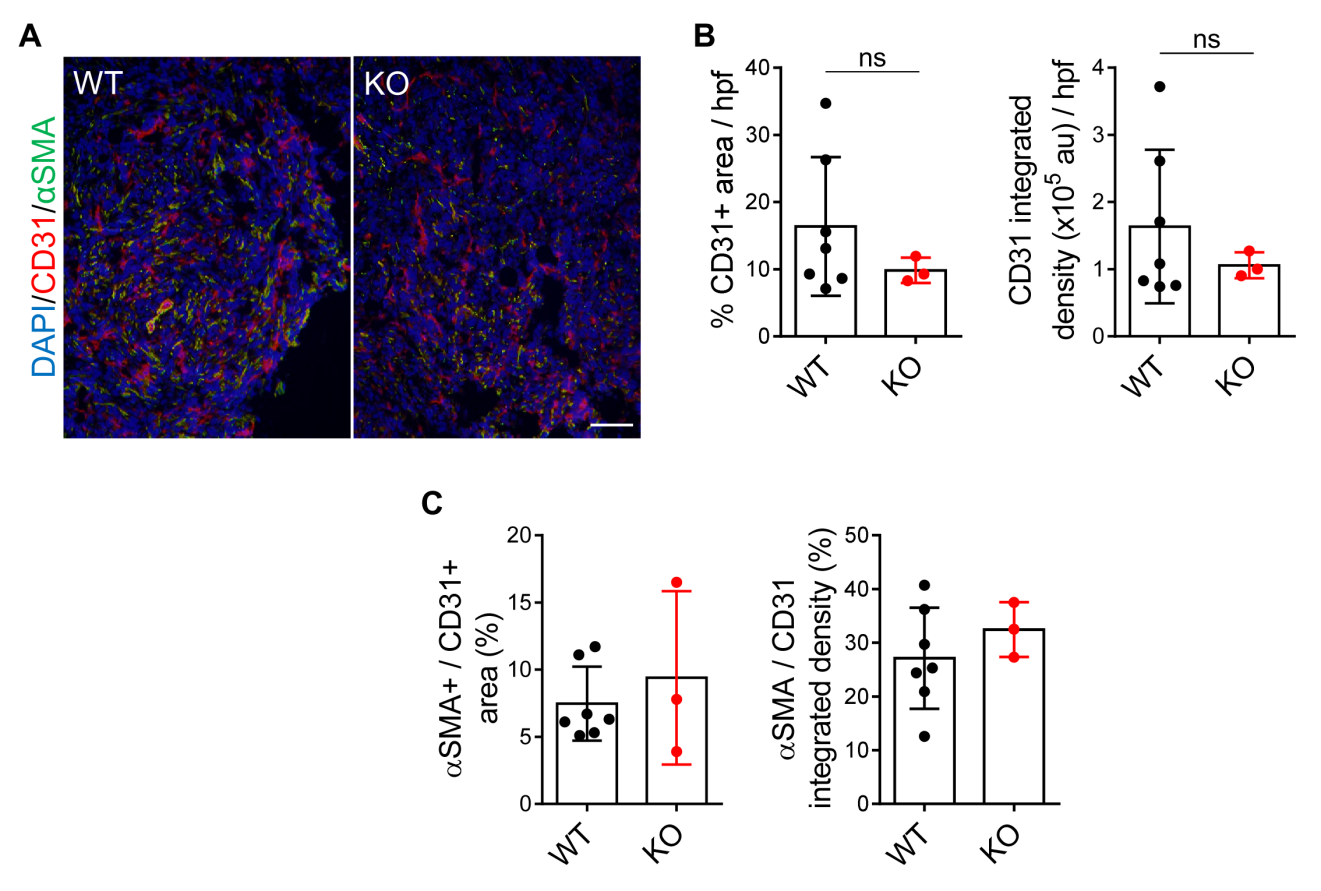
Tumor vascularity and pericyte coverage are not significantly different in ephrin-A1-deficient hosts. ( **A**) Representative images of CD31 (red), αSMA (green), and DAPI (blue) staining on cryosections of 4T1 primary tumors harvested from WT and KO littermates at day 14 post-implantation. Scale bar: 100 µm. ( **B**) Quantification of CD31+ area and integrated intensity in arbitrary units (au) per high power field (hpf) of view. ( **C**) Quantification of αSMA colocalization with CD31 as a percentage of αSMA+ over CD31+ stained area and integrated density. Data shown are averages ± SD with each data point representing an individual mouse ( *n*=3–7 mice per group).

To evaluate tumor vascularity and vessel function, we co-stained cryosections of 4T1 primary tumors from *Efna1* ^+/+^ and *Efna1* ^-/-^ littermates with CD31 and αSMA, markers for endothelial cells and pericytes, respectively. Colocalization of αSMA with CD31 acts as an indicator for functional endothelium within tumors. Surprisingly, we did not observe a change in CD31+ area or intensity in 4T1 tumors from *Efna1* ^+/+^ and *Efna1* ^-/-^ littermates ( Figure 4A, B). Furthermore, pericyte coverage on tumor vessels remained the same in tumors from *Efna1* ^+/+^ and *Efna1* ^-/-^ littermates ( Figure 4A, C). Together, these data suggest that loss of ephrin-A1 in the host does not affect tumor vessel formation and function in the primary tumor. *Underlying data* are available ^72^.

### Ephrin-A1-deficient lung microenvironment provides a less favorable metastatic niche

Our results from analysis of the immune infiltrate and vasculature of primary tumors, coupled with the significant difference in experimental lung metastasis between *Efna1* ^+/+^ and *Efna1* ^-/-^ mice, suggest that host factors critical to this metastatic phenotype are more likely to lie downstream of the primary tumor site. These steps include tumor cell trafficking to the lung vasculature, extravasation, and adaptation to new selective pressures of the lung microenvironment. 4T1 cell trafficking to the lung was not significantly different between *Efna1* ^+/+^ and *Efna1* ^-/-^ littermates after tail vein injections ( Figure 2A, B). Thus, we aimed to evaluate extravasation and adaptation to the lung metastatic niche in *Efna1* ^+/+^ and *Efna1* ^-/-^ hosts.

**Figure 5.**
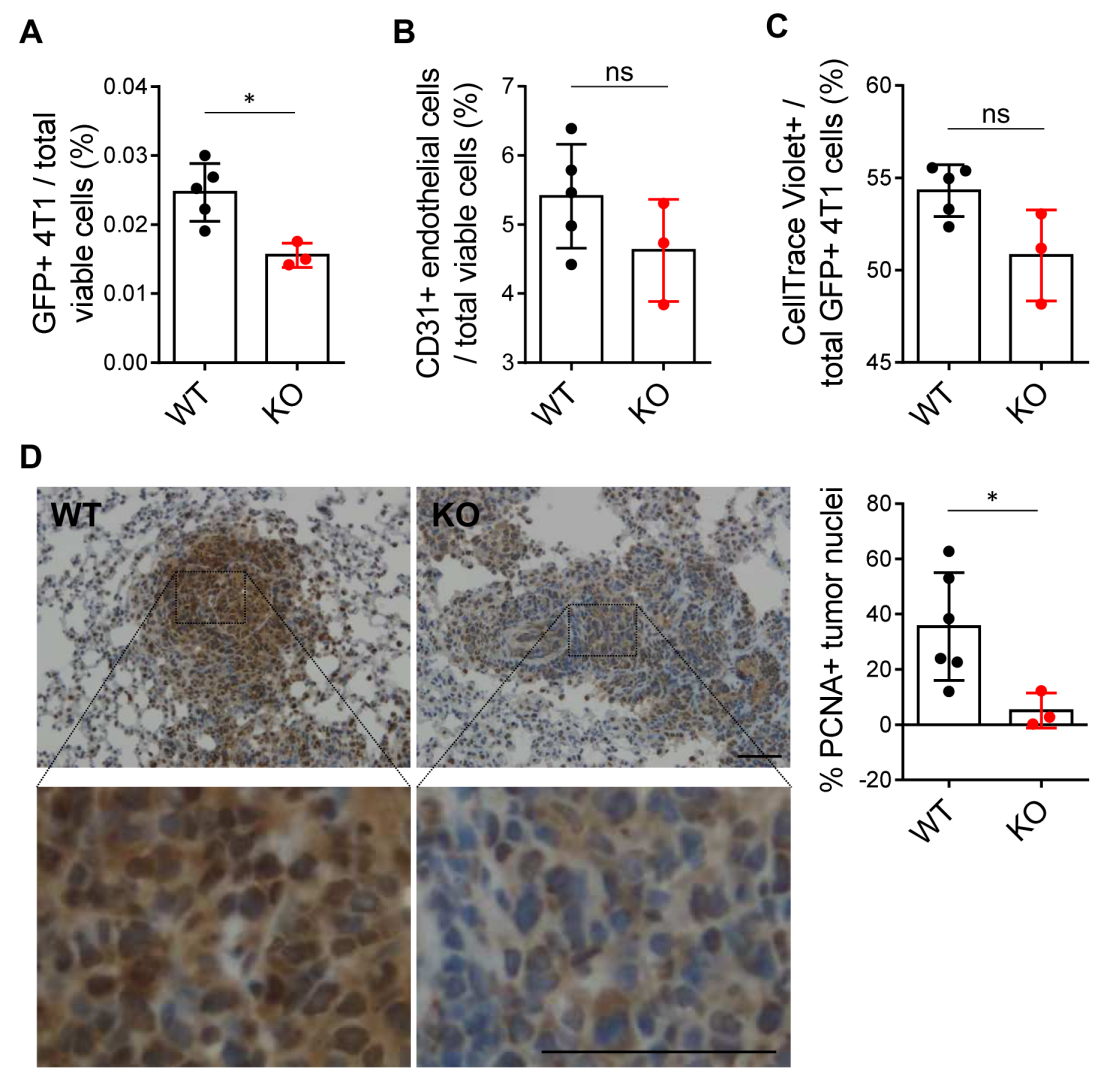
Ephrin-A1-deficient lung microenvironment provides a less favorable metastatic niche. ( **A**) Quantification of GFP+ 4T1 cells and ( **B**) CD31+ endothelial cells by flow cytometry in perfused lungs harvested from WT and KO littermates 24 hours post-tail vein injection. ( **C**) Percentage of GFP+ 4T1 cells in perfused WT and KO lungs that still contained CellTrace Violet dye, indicating reduced proliferation. ( **D**) Representative images of PCNA staining on FFPE sections of tumor-bearing lungs from WT and KO littermates 17 days after tail vein injection of 4T1-GFP-luciferase cells. Higher magnification images and blinded quantification of PCNA+ tumor cell nuclei shown. Scale bar: 50 µm. Data shown are averages ± SD with each data point representing an individual mouse ( *n*=3–6 mice per group). * *p*<0.05 (unpaired Mann-Whitney *U*-test).

To test extravasation of 4T1 cells *in vivo*, we injected 4T1-GFP-luciferase cells labeled with CellTrace Violet dye into the tail veins of *Efna1* ^+/+^ and *Efna1* ^-/-^ littermates. This dye is retained only in the labeled tumor cells and diminished after subsequent cell divisions, enabling quantification of short-term cell proliferation. At 24 hours after injection, we perfused the lungs with PBS to flush out remaining cells in the pulmonary vasculature and processed the lungs for flow cytometry. Decreased GFP+ 4T1 cells were found in ephrin-A1-deficient lungs compared to wild-type controls ( Figure 5A), suggesting that fewer cancer cells had extravasated into the lung parenchyma at this timepoint. This result may be partly due to decreased vascularity of ephrin-A1-deficient lungs at baseline. The percentage of CD31+ endothelial cells was slightly lower in knockout lungs but not significantly so with this sample size ( Figure 5B). Moreover, this was not due to decreased proliferation of the 4T1 cells within the 24-hour timeframe, as the amount of retained CellTrace Violet dye was not higher in 4T1 cells that had extravasated in knockout lungs compared to wild-type lungs ( Figure 5C). Together, these data suggest that extravasation of 4T1 cells is inhibited in knockout mice, compared to wild-type controls, and ephrin-A1 deficiency in the host lung may play a role in this process.

While decreased extravasation of tumor cells may explain in part the decreased lung metastases in *Efna1* ^-/-^ mice, another possibility is that once tumor cells have extravasated and established in the lung, they have reduced fitness of survival in ephrin-A1-deficient lungs, compared to wild-type lungs. There are many stressors in the lung metastatic niche that could impact the adaptability of the tumor cell. We used tumor proliferation index as a marker to evaluate how well tumor cells have adapted to a metastatic niche. Since no differences were observed in proliferation of 4T1 cells that had newly extravasated into the lung parenchyma of *Efna1* ^+/+^ and *Efna1* ^-/-^ littermates within the short 24-hour timeframe ( Figure 5C), we next assessed proliferation of tumor cells in lung micrometastases that had established over 17 days after tail vein injection ( Figure 2). There was a significant decrease in cell proliferation in metastatic foci established in *Efna1* ^-/-^ mice, compared to *Efna1* ^+/+^ controls, as indicated by PCNA staining ( Figure 5D). These findings suggest that reduced tumor cell lung colonization in *Efna1* ^-/-^ hosts is due to both decreased extravasation of cancer cells and decreased proliferation in the metastatic niche. *Underlying data* are available ^73, 74^.

## Discussion

In conclusion, host deficiency in ephrin-A1 inhibits metastasis by providing a less hospitable metastatic niche for cancer cell extravasation and colonization of the lung. Our data from 4T1 primary tumor specimens demonstrated no differences in primary tumor growth, infiltrating immune cell populations, and vascularity. This led us to investigate the metastatic process downstream from the primary tumors. We then found that lung colonization in knockout mice was decreased compared to wild-type mice as early as 24 hours post-tail vein injection of 4T1 cells, in part due to decreased extravasation. Moreover, the metastases that established in *Efna1* ^-/-^ lungs were not only reduced in number but also less proliferative compared to those in wild-type lungs. These studies offer insight on how host expression of ephrin-A1 may impact tumor growth and dissemination, but they also lead to additional questions.

Our ephrin-A1 knockout model is not tissue-specific nor inducible, which creates challenges in identifying specific mechanisms that contribute to our observed phenotype. For example, ephrin-A1 is highly expressed in embryonic stages of development and plays a known role in neuronal and mammary development ^66, 75– 79^. The transcriptional and epigenetic changes that occur *in utero* and during early physiological development as a result of ephrin-A1 deficiency in various tissues may all contribute to the observed phenotype; however, dissecting which changes are directly downstream of ephrin-A1 and critical to metastasis may be quite difficult. This challenge is further augmented when we consider the many cell types that can express ephrin-A1, especially immune, endothelial, and epithelial cells. Additionally, ephrin-A1 on these cell types presumably interacts with EphA receptors on various stromal and tumor cells. In the absence of host ephrin-A1, forward signaling in these EphA receptors may be reduced, or it may be conserved through compensatory interaction with other ephrin-A ligands. If other ephrin-A1 ligands do not compensate for the lack of ephrin-A1, perhaps EphA receptors in these cells are available for more ligand-independent signaling. These are all reasonable hypotheses that may be supported with more molecular and biochemistry studies.

Many studies have demonstrated ephrin-A1’s role in immune cell adhesion and migration. Although we did not observe significant differences in tumor immune infiltrate, this does not preclude a role for ephrin-A1 in these cell populations. Immune cells engage in a complex network of crosstalk, and it is possible that loss of ephrin-A1 in one cell type may mask the effects it has in another. One intriguing difference we observed was an increase in macrophages in *Efna1* ^-/-^ tumor-bearing lungs. However, we have not determined if this difference occurs in the specific context of a stressor, such as tumor metastasis, or if knockout mice have increased macrophages at baseline. Because ephrin-A1 has been shown to impact monocyte chemotaxis and adhesion to the endothelium, it is reasonable to hypothesize that ephrin-A1 may affect recruitment of monocytes from circulation into lung tissue where they differentiate into macrophages. Macrophages in the lung are known to play a role in forming the pre-metastatic niche and maintaining a metastatic niche ^80^. Though we demonstrate increased macrophages in ephrin-A1-deficient lungs, it remains to be seen if these macrophages are polarized towards an anti-tumor or a pro-tumor response. Nevertheless, this offers evidence of a novel role of ephrin-A1 in macrophage recruitment, differentiation, or survival, which requires further investigation.

In addition, ephrin-A1 has been shown to regulate expression of adhesion molecules on endothelial cells and promote angiogenesis. Modulation of surface expression of adhesion proteins, such as ICAM-1 and VCAM-1, on endothelial cells impact binding to immune cells and cancer cells ^8, 40^. Thus, it is possible that ephrin-A1 on endothelial cells may mediate cancer cell transendothelial migration through modulation of these adhesion proteins. While this result may be consistent with published literature, in contrast to ephrin-A1’s known role in angiogenesis, we did not observe differences in angiogenesis between tumors from *Efna1* ^+/+^ and *Efna1* ^-/-^ hosts. This discrepancy may be due to a couple reasons. First, most studies reporting on ephrin-A1’s impact on angiogenesis have shown its effect through EphA receptor signaling on the endothelial cell, not necessarily through ephrin-A1 directly in the endothelium ^66– 69^. Loss of ephrin-A1 in the endothelium and other host tissues is unlikely to completely abrogate EphA receptor signaling in the endothelium, as other ephrin ligands are able to promiscuously bind to the same EphA receptors and may even compensate for the loss of ephrin-A1 ^37^. Second, some of these studies use soluble ephrin-A1, instead of membrane-bound or cell-surface ephrin-A1. In our *Efna1* ^-/-^ model, both cell-surface, membrane-bound ephrin-A1 and soluble, secreted ephrin-A1 are lost *in vivo*, and these two forms of ephrin-A1 have been shown to have competing effects ^13^.

The different forms of ephrin-A1, as well as the range of interactions with various Eph receptors, show how potentially complex the molecular mechanisms can be when considering host deficiency of ephrin-A1. A clue into this complicated investigation can be found in our data obtained with ephrin-A1 heterozygote controls. When comparing tumor metastasis and immune infiltrate, results from *Efna1* ^+/-^ mice were much more comparable to wild-type than knockout littermate controls. This suggests that ephrin-A1 has a genetically dominant effect – one wild-type allele may be sufficient to induce the wild-type phenotype.

Although we focused our inquiries on primary mammary tumors and lung metastases, there is much more to be explored. 4T1 cells, like human breast cancer, metastasize to other organ sites, such as the bone, liver, and brain. The lungs in *Efna1* ^-/-^ hosts may or may not be the only organ that provides a less favorable environment for colonizing tumor cells than those in *Efna1* ^+/+^ hosts. We observed differences in recurrent primary tumor, in addition to lung metastases, which may indicate that tumor cell apoptosis or senescence is altered in knockout hosts. If this is the case, one may infer that primary tumors should also be smaller in knockout mice. Although we did not observe differences in primary tumors, it is possible that the number of 4T1 cells that were injected and the amount of Matrigel used to implant these cells, though small, may have obscured these results.

While much of the published literature on ephrin-A1 focuses on its tumor suppressive role in the tumor cell, this novel study demonstrates that its role in the host tissues may be tumor-promoting. This suggests that the function of ephrin-A1is cell type-dependent and that if there is a way to target ephrin-A1 in host tissues, rather than in the tumor, targeting host ephrin-A1 to inhibit metastasis may be a strategy worth considering. Further elucidating the mechanisms by which ephrin-A1 in host cells impact cancer relapse and metastasis may enhance our understanding of the metastatic process and ultimately shed new light on novel therapeutic strategies.

## Data availability

### Underlying data

Harvard Dataverse: Host deficiency in ephrin-A1 inhibits breast cancer metastasis; https://dataverse.harvard.edu/dataverse/hostEfna1metastasis.

This project contains the following underlying data:

Harvard Dataverse: 4T1 primary tumor dimensions and weights. https://doi.org/10.7910/DVN/AGKDWV ^46^. (4T1 primary tumor dimensions from digital caliper measurements, volume calculations, and weights (related to Figure 1A, C, D.)

Harvard Dataverse: 4T1 recurrent primary and spontaneous lung metastases. https://doi.org/10.7910/DVN/FU8JEY ^47^. (Spontaneous 4T1 lung metastases quantification and recurrent primary tumor weights (related to Figure 1E, F.)

Harvard Dataverse: Images and quantification of 4T1-GFP-luciferase experimental lung metastases. https://doi.org/10.7910/DVN/2ANDYX ^48^. (Experimental 4T1-GFP-luciferase lung metastases quantification and images (related to Figure 2C–E.))

Harvard Dataverse: 4T1-GFP-luciferase bioluminescence images and quantification post-tail vein injection. https://doi.org/10.7910/DVN/39D0YR ^49^. (4T1-GFP-luciferase bioluminescence quantification and images (related to Figure 2A, B).)

Harvard Dataverse: 4T1 primary tumor flow cytometry. https://doi.org/10.7910/DVN/ZRX2RG ^61^. (Flow cytometry files (fcs), gating and analysis (wsp), and panels (xlsx) containing immune profiling of 4T1 primary mammary tumors from *Efna1* ^+/+^, *Efna1* ^+/-^, and *Efna1* ^-/-^ littermate mice (related to Figure 3A, B))

Harvard Dataverse: 4T1-GFP-luciferase tumor-bearing lung flow cytometry. https://doi.org/10.7910/DVN/S06NQ1 ^62^. (Flow cytometry files (fcs), gating and analysis (wsp), and panels (xlsx) containing immune profiling of 4T1-GFP-luciferase tumor-bearing lungs from *Efna1* ^+/+^, *Efna1* ^+/-^, and *Efna1* ^-/-^ littermate mice (related to Figure 2D, 3C, D).)

Harvard Dataverse: 4T1-GFP-luciferase 24-hr lung colonization flow cytometry. https://doi.org/10.7910/DVN/G7TAAE ^73^. (Flow cytometry files (fcs), gating and analysis (wsp), and panels (xlsx) containing profiling of 24-hr 4T1-GFP-luciferase tail vein injected lungs from *Efna1* ^+/+^ and *Efna1* ^-/-^ littermate mice (related to Figure 5A–C).)

Harvard Dataverse: CD31 and aSMA images and quantification of 4T1 primary tumors. https://doi.org/10.7910/DVN/MOYPE7 ^72^. (4T1 primary tumor CD31 and αSMA staining quantification and images (related to Figure 4A–C).)

Harvard Dataverse: PCNA images and quantification of 4T1-GFP-luciferase lung metastases. https://doi.org/10.7910/DVN/8AJKFM ^74^. (Lung metastasis PCNA staining quantification and images (related to Figure 5D).)

Data are available under the terms of the Creative Commons Zero “No rights reserved” data waiver (CC0 1.0 Public domain dedication).
