# Supplementary material for: Host deficiency in ephrin-A1 inhibits breast cancer metastasis
Source: F1000Res. 2020 May 14;9:217. Originally published 2020 Mar 30. [Version 2] doi: 10.12688/f1000research.22689.2 (PMC7194498; doi:10.12688/f1000research.22689.2)
Supplement: Peer review history

# Peer review: Reviewer response for version 2

**DOI:** 10.5256/f1000research.26469.r63416

**Published:** 2020-05-22

**Authors:** Nikki Cheng

**Affiliations:** 1 Department of Pathology and Laboratory Medicine, University of Kansas Medical Center (KUMC), Kansas City, KS, USA

Authors have sufficiently addressed concerns.

Is the work clearly and accurately presented and does it cite the current literature?

Yes

If applicable, is the statistical analysis and its interpretation appropriate?

Yes

Are all the source data underlying the results available to ensure full reproducibility?

No source data required

Is the study design appropriate and is the work technically sound?

Yes

Are the conclusions drawn adequately supported by the results?

Yes

Are sufficient details of methods and analysis provided to allow replication by others?

Yes

Reviewer Expertise:

NA

I confirm that I have read this submission and believe that I have an appropriate level of expertise to confirm that it is of an acceptable scientific standard.

# Peer review: Reviewer response for version 1

**DOI:** 10.5256/f1000research.25051.r61807

**Published:** 2020-04-22

**Authors:** Nikki Cheng

**Affiliations:** 1 Department of Pathology and Laboratory Medicine, University of Kansas Medical Center (KUMC), Kansas City, KS, USA

EphrinA1 is a cell surface bound protein that has been shown to be tumor suppressive when expressed in cancer cells. In this study, the authors characterize the effects of the microenvironment on mammary tumor progression using variations of the 4T1 mammary tumor model- spontaneous, and experimental metastasis. By injecting 4T1 cells in ephrinA1 knockout mice, they show that ephrinA1 knockout does not affect primary tumor growth and inhibits metastasis and recurrence. The study is generally well written, and the phenotypes are interesting. Comments are described below:

1. The discussion could be better expanded.
2. How do expression of ephrinA1 and corresponding receptors in the stroma of primary tumor vs. lung tissue compare in normal and tumor bearing mice? How do results fit into the bigger picture of what is known about this?
3. Recommend expanding on the discussion of the mechanism regarding ephrinA1 mediated lung metastasis. In particular:                                            

              a) How might ephrinA1 signaling might affect macrophage function in the lung.

               b) Please expand on what is known about macrophage polarization, effects on T cells in the lung. How would ephrinA1 signaling affect macrophage function in the formation of pre-metastatic vs. metastatic niche?

               c) How would ephrinA1 ko affect Eph receptorA1 vs. A2 signaling in the lung stroma?

               d) Further discussion the clinical implications of the study would be helpful, especially since the tumor suppressive/oncogenic effects of ephrinA1 appear to be cell type dependent.

More minor comments:

1. Figure 1A, C, D, Figure 3, Figure 4, Figure 5B-C: Statistics are not consistently shown. Even if data show no statistical significance, it would be helpful to include p- value or indicate n.s to indicate the analysis was done.
2. Figure 4: CO-IF staining is a bit difficult to see. It would be helpful to show magnified insets for clearer details.
3. Figure 5: recommend revising figure legend. From the way the figure legend is stated “ **Ephrin-A1-deficient lung microenvironment provides a less favorable metastatic niche**,” I would expect data correlating decreased metastasis with changes in the lung microenvironment of ephinA1 KO mice. However, this is not the case - no changes in endothelial cells were observed. Alternatively, for Figures 3 and 5 - recommend reorganizing some of the data so that the messages are clearer. Figure 3 figure legend indicates that “ **Tumor-infiltrating immune populations are not significantly different in ephrin-A1-deficient hosts” **However, Figure 3D show increased in macrophage recruitment to lung tissues in ephrinA1 KO mice. This data would be more appropriate in Figure 5, which shows decreased lung metastasis, along with characterization of endothelial cells in the lung.

Is the work clearly and accurately presented and does it cite the current literature?

Yes

If applicable, is the statistical analysis and its interpretation appropriate?

Yes

Are all the source data underlying the results available to ensure full reproducibility?

No source data required

Is the study design appropriate and is the work technically sound?

Yes

Are the conclusions drawn adequately supported by the results?

Yes

Are sufficient details of methods and analysis provided to allow replication by others?

Yes

Reviewer Expertise:

NA

I confirm that I have read this submission and believe that I have an appropriate level of expertise to confirm that it is of an acceptable scientific standard.

# Response

**Published:** 2020-05-07

**Authors:** Eileen Shiuan

**Affiliations:** Vanderbilt University, USA

Thank you for reviewing this manuscript! We have addressed your suggestions and comments with the following revisions:

1) Expanded discussion on potential impact of ephrin-A1 deficiency on other ephrin-A1 ligands and EphA receptors in stromal cells and their signaling.

2) Expanded discussion on what is known about the role of macrophages in the lung metastatic niche and how ephrin-A1 deficiency may impact recruitment and function of macrophages.

3) Expanded discussion of clinical implications of the study.

4) Indicated "ns" on graphs where data from WT and KO showed a trending difference that was not statistically significant.

5) Minor text changes.

# Peer review: Reviewer response for version 1

**DOI:** 10.5256/f1000research.25051.r61809

**Published:** 2020-04-15

**Authors:** Elena B. Pasquale

**Affiliations:** 1 Tumor Initiation and Maintenance Program, NCI-Designated Cancer Center, Sanford Burnham Prebys Medical Discovery Institute, La Jolla, CA, USA

This is a novel and interesting study on the role of ephrin-A1 expressed in the host in breast cancer lung metastasis. The role of ephrin-A1 in the tumor microenvironment and the metastatic niche is poorly understood. This manuscript addresses this gap in knowledge by systematically investigating how ephrin-A1 expressed in tumor immune cells, the tumor vasculature, the lung vasculature and the lung microenvironment affects lung metastases formed by 4T1 mouse breast cancer cells. These studies uncover a new role of host ephrin-A1 in promoting the formation of lung metastases by breast cancer cells and in tumor recurrence.

 Overall, the manuscript is of high quality and most conclusions are well supported by the data presented. I only have several suggestions that the authors might want to consider.

 The authors conclude that host ephrin-A1 does not affect the growth of the primary tumor. In contrast, they find that ephrin-A1 present in the microenvironment promotes primary tumor recurrence as well as the growth of lung metastases. However, the primary tumors were seeded by injecting tumor cells mixed with Matrigel. I wonder whether the use of Matrigel might create an artificial microenvironment that obfuscates possible effects of host ephrin-A1 on the primary tumor, particularly at early stages of tumor development. The authors should indicate in the Methods the volume of Matrigel used and mention also in the Results that the tumor cells were injected mixed with Matrigel. They could also briefly mention in the text the possible effects of Matrigel on primary tumor development (or discuss why they believe using Matrigel is not a significant issue).

 In some instances (such as Fig. 3A, dendritic cells panel, and possibly Fig. 5B) the authors find that there is no statistically significant difference between mice with different genotypes. However, n (presumably indicating the number of mice) is small in some groups and the variability of the data is high, so it could be argued that analysis of more mice could yield a more definitive result. Perhaps the conclusions could be stated more cautiously in these cases.

 On page 3, left column, third paragraph, ref. 43 does not report a tumor suppressive role of ephrin-A1 but rather the opposite.

 Very minor points:

- Animal models section in the Methods: In a couple of sentences, it would seem better to say “female littermates” rather than “gender-matched littermates” since only females were used.
- Specify in the figure legends for the histograms whether the dots represent the number of mice.
- Page 10, right column, 3 lines from the bottom. “Higher” would be better than “increased”.
- Page 11, right column, second paragraph, line 13. It should be “have” not “has”.

Is the work clearly and accurately presented and does it cite the current literature?

Yes

If applicable, is the statistical analysis and its interpretation appropriate?

Yes

Are all the source data underlying the results available to ensure full reproducibility?

Yes

Is the study design appropriate and is the work technically sound?

Yes

Are the conclusions drawn adequately supported by the results?

Partly

Are sufficient details of methods and analysis provided to allow replication by others?

Yes

Reviewer Expertise:

Signal transduction, with focus on Eph receptors and ephrins in cancer and the nervous system.

I confirm that I have read this submission and believe that I have an appropriate level of expertise to confirm that it is of an acceptable scientific standard.

# Response

**Published:** 2020-05-07

**Authors:** Eileen Shiuan

**Affiliations:** Vanderbilt University, USA

Thank you for reviewing this manuscript! We have addressed your suggestions and comments with the following revisions:

1) Added details regarding use of Matrigel in the Methods and Results and discussed potential confounding effects of Matrigel on primary tumor volume in the Discussion.

2) For data with small sample sizes showing a nonsignificant trend, revised interpretation of data to include possibility of significant differences if more mice were used.

3) Indicated in all figure legends that each data point corresponds to an individual mouse.

4) Minor text changes.

# Peer review: Reviewer response for version 1

**DOI:** 10.5256/f1000research.25051.r61805

**Published:** 2020-04-07

**Authors:** Jinming Yang

**Affiliations:** 1 Department of Cancer Biology and Toxicology, College of Medicine, Markey Cancer Center, University of Kentucky, Lexington, KY, USA

The role of the ephrin-A1/EphA2 signaling axis in tumor cells has been studied extensively. However, many cell types in the tumor microenvironment also express ephrin/Eph molecules, the functions of which are not completely understood.  This report investigates the EphA2/ephrin-A1 axis-mediated host-tumor interaction. By implanting tumors in wild-type or ephrin-A1 knockout hosts, the authors showed that loss of ephrin-A1 in tumor microenvironment significantly reduced tumor metastasis, both in the spontaneous allograft model and in experimental metastasis assays via tail vein injection.

 Overall, the studies are well designed and the manuscript is well written. Using both spontaneous metastasis and tail vein injection models increases the rigor and reproducibility of the studies. Experiments are also well controlled by using littermates and scoring blindly. The authors showed negative findings in tumor-infiltrating immune populations and tumor vascularity between ephrin-A1 knockout and wild-type control littermates. Nevertheless, the exact molecular mechanism of ephrin-A1 in tumor metastasis has not been determined. For example, decreased lung metastatic lesions could be due to reduced survival of tumor cells in circulation, extravasation into lung, or growth in the lung microenvironment, as ephrin-A1 is expressed in the lung epithelium, vascular endothelial cells, and some of immune cells. Because this is a global knockout, dissecting specific roles of ephrin-A1 in each cell population in the tumor microenvironment are challenging. After all, the fact that ephrin-A1 plays a critical role in metastasis should be of interest to the general audience in the field of cancer research.

Is the work clearly and accurately presented and does it cite the current literature?

Yes

If applicable, is the statistical analysis and its interpretation appropriate?

Yes

Are all the source data underlying the results available to ensure full reproducibility?

Yes

Is the study design appropriate and is the work technically sound?

Yes

Are the conclusions drawn adequately supported by the results?

Yes

Are sufficient details of methods and analysis provided to allow replication by others?

Yes

Reviewer Expertise:

NA

I confirm that I have read this submission and believe that I have an appropriate level of expertise to confirm that it is of an acceptable scientific standard.

# Response

**Published:** 2020-05-07

**Authors:** Eileen Shiuan

**Affiliations:** Vanderbilt University, USA

Thank you for reviewing this manuscript!
